# Combination Delivery of Alpha-Tocopheryl Succinate and Curcumin Using a GSH-Sensitive Micelle (PAH-SS-PLGA) to Treat Pancreatic Cancer

**DOI:** 10.3390/pharmaceutics12080778

**Published:** 2020-08-16

**Authors:** Tilahun Ayane Debele, Hung-Chang Wu, Shang-Rung Wu, Yan-Shen Shan, Wen-Pin Su

**Affiliations:** 1 Institute of Clinical Medicine, College of Medicine, National Cheng Kung University, No.138, Sheng Li Road, Tainan 704, Taiwan; z10803012@ncku.edu.tw (T.A.D.); ysshan@mail.ncku.edu.tw (Y.-S.S.); 2 Department of Internal Medicine, Chi Mei Medical Center, Tainan 710, Taiwan; hungchang.wu@gmail.com; 3 Institute of Basic Medical Sciences, College of Medicine, National Cheng Kung University, Tainan 704, Taiwan; z10208056@ncku.edu.tw; 4 Department of Dentistry & Institute of Oral Medicine, College of Medicine, National Cheng Kung University, Tainan 704, Taiwan; 5 Department of Surgery, National Cheng Kung University Hospital, College of Medicine, National Cheng Kung University, Tainan 704, Taiwan; 6 Departments of Oncology and Internal Medicine, National Cheng Kung University Hospital, College of Medicine, National Cheng Kung University, Tainan 704, Taiwan

**Keywords:** GSH-sensitive micelle, poly(allylamine hydrochloride) (PAH), poly(lactic-co-glycolic acid) (PLGA), alpha-tocopheryl succinate, curcumin

## Abstract

Pancreatic cancer is one of the highest causes of mortality throughout the world; thus, it requires an effective treatment strategy. Some chemotherapeutic agents used in the clinics or under clinical trials are hydrophobic and have poor aqueous solubility; consequently, they also have minimal systemic bioavailability. Nanoparticle-based drug delivery tactics have the potential for overcoming these limitations and enhancing their therapeutic efficacy. Herein, a glutathione (GSH)-sensitive micelle (PAH-SS-PLGA) was synthesized for the combined delivery of alpha-tocopheryl succinate (TOS) and curcumin to improve its therapeutic efficacy. The chemical structures of PAH-SS-PLGA were analyzed using Proton Nuclear Magnetic Resonance (^1^H-NMR) and Fourier Transform Infrared (FTIR) spectroscopy, whereas the particle size, zeta potential, and surface morphology were observed using dynamic light scattering (DLS) and transmission electron microscopy (TEM). In vitro drug release results revealed that more TOS and curcumin were released in the presence of GSH (5 mM) than the physiological pH value. Fluorescence microscopy images revealed that nanoformulated curcumin/rhodamine was uptaken by PAN02 pancreatic cancer cells. In vitro cytotoxicity assays showed higher cytotoxicity for nanoformulated TOS and/or curcumin than free TOS and/or curcumin. In addition, higher cytotoxicity was observed for combination drugs than free drugs alone. Most interestingly, at all tested concentrations of nanoformulated drugs (PAH-SS-PLGA, TOS, and curcumin), the calculated combination index (CI) value was less than one, which shows that TOS and curcumin have a synergistic effect on cellular proliferation inhibition. Overall, synthesized co-polymers are the best carriers for combination drugs, TOS, and curcumin, because they enhance the therapeutic efficacy and improve pancreatic cancer treatments.

## 1. Introduction

Nowadays, several targeted stimuli-sensitive nanocarrier-based drug delivery systems are of great interest for the treatment of cancers [1,2]. Nanocarriers enhance prolonged systemic circulation, improve the solubility of hydrophobic drugs, enhance metabolic stability, prevent premature release, and target delivery to the site of interest to minimize the systemic side effects associated with conventional drug formulations [2,3,4]. Of these, polymeric micelles are widely explored for their potential applications in the drug delivery field [5]. Polymeric micelles are synthesized from amphiphilic copolymers, which have the capacity to self-assemble to form micelles with a core/shell structure in an aqueous milieu [6]. The prominent features of such micelles include the capacity to encapsulate hydrophobic drugs into their core region and increase the solubility of lipophilic drugs, which in turn significantly enhance therapeutic efficacy [7]. Moreover, stimuli-sensitive segments are inserted as the linker, which enable the nanocarriers to release their cargo at the site of interest in response to specific external or internal stimuli, which trigger the release mechanism [8]. Of these, Reduced glutathione (GSH)-sensitive polymeric micelles are widely studied due to intrinsic differences between various solid tumors and the surrounding normal tissues in terms of their concentration of excess GSH levels in the cancer cells relative to the normal cells [9,10,11,12,13]. Several researchers have postulated that a high intracellular GSH concentration (1–10 mM) facilitates the degradation of disulfide linkages after cellular uptake to release their cargo, whereas synthesized materials are stable in the extracellular environment due to low concentrations of GSH (1–10 μM) [14]. As the result, once the GSH-sensitive micelles are taken up by cancer cells, disulfide bonds are cleaved, the micelles disassemble, and drugs are released in the cytosol. By taking this into consideration, several researchers have synthesized GSH-sensitive micelles using biocompatible polymers [15].

Poly(allylamine hydrochloride) (PAH) is a cationic water-soluble biodegradable synthetic polymer that has been used for several applications in the biomedical field. It is one of the most frequently studied cationic polyelectrolytes in the preparation of ultrathin polyelectrolyte multilayers using a layer-by-layer method [16]. Furthermore, cationic polymers (such as polyethyleneimine, polylysine, polyhistidine, and PAH) are widely explored for the drug delivery systems [17,18,19,20]. However, the primary limitation of the PAH polymer in the drug delivery is its high cytotoxicity to the normal cells due to its primary amines along the polymer backbone [21]. It is expected that surface modification of the PAH polymer can minimize the PAH-related cytotoxicity. It was reported that guanidinylation, glycolylation, or imidazolyl substitution of the amino groups of PAH results in a significant reduction of its cytotoxicity [22,23]. Similarly, a different hydrophobic polymer is conjugated with the PAH amine groups, and the modified polymer tends to self-assemble in aqueous solution to form micelles due to its amphiphilic nature. This study focuses on improving the biological properties of PAH by its modification with the citraconic anhydride and further by conjugating with Poly(lactic-co-glycolic acid) (PLGA) to form a biocompatible micelle for drug delivery systems.

PLGA is the most common biocompatible and biodegradable polymer, and it is extensively used in the drug delivery systems [24]. PLGA has tunable mechanical properties, and most importantly, is an FDA-approved polymer. Herein, poly(allylamine)-citraconic anhydride (PAH-Cit) was conjugated with the cystamine-derivetized PLGA (PLGA-Cys) to form a stable GSH-sensitive copolymer (PAH-SS-PLGA). Due to its amphiphilic nature, the synthesized copolymer is an excellent candidate for the encapsulation of hydrophobic drugs, such as tocopheryl succinate (TOS) and curcumin, in their core regions.

D-α-tocopheryl succinate is the most useful form of vitamin E derivative to inhibit cancer proliferation by inducing mitochondria dysfunction and apoptosis with low/no cytotoxicity to the normal cells [25,26]. In addition, the synergistic therapeutic effects of TOS with chemotherapeutics drugs were widely explored both in the in vitro and in vivo studies.

Curcumin, the principal bioactive component of *Curcuma longa* (turmeric), is a widely explored a natural polyphenolic compound. Different research findings have shown that curcumin has potent anti-proliferative, anti-metastatic, and anti-angiogenic effects both in cell culture and animal studies [27,28,29]. Although curcumin has shown significant efficacy in cell culture studies, it has limited efficacy in clinical studies, which is maybe due to its poor water solubility and poor bioavailability [30,31]. These barriers can be overwhelmed by using nanocarriers. Therefore, we synthesized a biocompatible polymeric micelle to encapsulate both TOS and curcumin in the core of micelles, which will improve its solubility, stability, bioavailability, and therapeutic efficacy.

In general, several techniques have been used to characterize the synthesized copolymers, including Proton Nuclear Magnetic Resonance (^1^H-NMR) and Fourier Transform Infrared (FTIR) spectroscopy, and UV-VIS. The physiochemical properties of synthesized micelles such as particle size, zeta potential, and surface morphology were assessed using dynamic light scattering (DLS) and TEM. The feasibility of synthesized micelles, such as drug-loading capacity, encapsulation efficiency, drug releases, and cellular uptake studies were investigated. In vitro cytotoxicity tests, such 3-(4,5-dimethylthiazol-2-yl)-2,5-diphenyltetrazolium bromide (MTT), colony, apoptosis, and cell cycle inhibition were performed for all precursors, free drugs, and nanoformulated drugs.

## 2. Methods and Materials

### 2.1. Materials

Poly (lactic-co-glycolic acid, PLGA, Mw 35.8 kDa), poly (allylamine hydrochloride) (PAH, Mw 17 kDa), citraconic anhydride, D-α-tocopheryl succinate, curcumin, N-hydroxy succinimide (NHS) and 1-ethyl-3-(3-dimethylaminopropyl) carbodiimide hydrochloride (EDC) were purchased from Sigma Aldrich Inc. (St. Louis, MO, USA). All the chemicals were analytical grade and were used without further purification. High-purity deionized water is used throughout the experiments.

### 2.2. Synthesis of Poly(allylamine)-Citraconic Anhydride Conjugated with the Poly(Lactic-co-Glycolic Acid)-Cystamine (PAH-SS-PLGA)

**Scheme 1:**
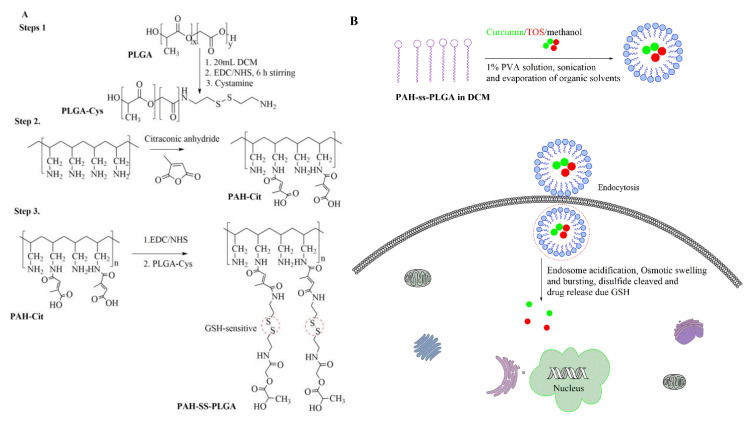
(**A**) Scheme of poly(allylamine)-citraconic anhydride-poly(lactic-co-glycolic acid)-cystamine (PAH-SS-PLGA) synthesis and (**B**) endocytosis-mediated uptake of curcumin/TOS-loaded micelle. TOS: tocopheryl succinate.

The poly(allylamine)-citraconic anhydride-poly(lactic-co-glycolic acid)-cystamine (PAH-SS-PLGA) conjugate was synthesized in three reaction steps as shown in Scheme 1, by simple modification of the previously reported protocol [32]. In the first reaction steps, one of the amine groups of cystamine reacted with the activated carboxyl group of PLGA to form poly(lactic-co-glycolic acid)-cystamine (PLGA-Cys). Briefly, PLGA (2 g, 0.0558 mmol) was dissolved in anhydrous dichloromethane (DCM, 20 mL) and activated by adding 10 times molar excess of EDC (0.107 g, 0.558 mmol) and NHS (0.0643 g, 0.558 mmol) under stirring for 6 h at room temperature. Then, 10 times molar excess of cystamine (0.126 g, 0.558 mmol) was added into the above reaction mixture in the presence of 51.2 μL Triethylamine (TEA) (2.5 mmol). The solution was stirred for 24 h at room temperature, and the reaction mixture was dialyzed for 96 h against distilled water using a dialysis bag (spectra/Por, MWCO: 12–14 kDa) to remove excess unreacted cystamine, other reactants, and by-products, within 6 h interval water exchange, and the final product (PLGA-Cys) was freeze dried, stored at 4 °C, and used without further treatment.

In the second reaction steps, poly(allylamine hydrochloride) (PAH) was reacted with the citraconic anhydride in the alkaline medium. Briefly, 1 g of PAH (10.69 mMole) was dissolved in 30 mL of NaOH (1 M) and stirred for 12 h. Then, three times molar excess of citraconic anhydride (32.08 mMole) was added into the PAH solution and the pH of the solution was maintained above 9.0 by using NaOH (6 M). After 24 h of reaction, the resultant mixture was dialyzed for 96 h against distilled water using a dialysis bag (spectra/Por, MWCO: 12–14 kDa) to remove excess unreacted citraconic anhydride, other reactants, and by-products, within a 6-h interval water exchange, and the final product (PAH-Cit) was freeze dried, stored at 4 °C, and used without further treatment. On the third reaction steps, the amino group of PLGA-Cys was reacted with the carboxyl group of PAH-Cit in the presence of EDC/NHS. Briefly, PAH-Cit (0.1 g) was dissolved in 15 mL of DMSO and then activated by EDC (0.042 g) and NHS (0.025 g) for 6 h under stirring. Then, PLGA-Cys (1 g) dissolved in 15 mL of DMSO was added into the above reaction mixture in the presence of 50 μL TEA, and the reaction was continued for 24 h under stirring, at room temperature. The reaction mixture was dialyzed for 96 h against distilled water using a dialysis bag (spectra/Por, MWCO: 20–25 KDa) to remove excess DMSO, unreacted mixture, and by-products, within a 6 h interval water exchange, and the final product (PAH-SS-PLGA) was freeze dried, stored at 4 °C, and used without further treatment. Chemical structures of PAH-Cit, PLGA-Cys, and PAH-SS-PLGA were characterized using ^1^H-NMR and FTIR. The infrared absorption spectra were collected from 4000 to 620 cm^–1^ for all precursors and synthesized co-polymers. The spectra were recorded on a Perkin Elmer spectrometer operating in the attenuated total reflection (ATR) mode, and 4 scans were performed for a resolution of 4 cm^–1^. For ^1^H-NMR analysis, PAH and PAH-Cit were dissolved in D_2_O, whereas PLGA-Cys and PAH-SS-PLGA were dissolved in DMSO-d6 and CDCl_3_, respectively and analyzed by a Bruker AVANCE 600 MHz NMR spectrometer (Varian, Inc., Palo Alto, CA, USA).

### 2.3. Preparation of Drug-Loaded and Drug-Free PAH-SS-PLGA Micelle

Curcumin/TOS-loaded micelles were prepared using the emulsion–solvent evaporation method as mentioned elsewhere by simple modification [33]. Briefly, 10 mg of curcumin, 20 mg of TOS and 40 mg of PAH-SS-PLGA were dissolved together in 10 mL of DCM. Next, the solution was emulsified in 10 mL of 1% PVA to form an oil-in-water emulsion. The emulsification was carried out using a probe type sonicator at 40% amp, (pulse 30 on and 15 off) for 5 min. The organic solvent was removed at 35 °C for 4 h, and the reaction continued, stirring overnight at room temperature to completely remove organic solvents. Then, the resulting suspension was centrifuged at 13,000 rpm for 20 min to remove any unencapsulated drugs, and the pellets were resuspended in 10 mL of distilled water or Phosphate buffered saline (PBS). The drug-loaded micelles were kept at 4 °C for further use. The single drug (i.e., TOS or curcumin)-loaded micelles and drug-free micelles were prepared using a similar procedure. The average hydrodynamic diameter of the empty micelles and drug-loaded micelles were determined by dynamic light scattering (Malvern Zetasizer Nano S apparatus equipped with a 4.0 mW laser operating at λ = 633 nm and at a scattering angle of 90°). The morphology of the empty micelles and drug-loaded micelles were analyzed using TEM (with a JEOL JEM-100CX-II instrument, JEOL, Tokyo, Japan) at a voltage of 120 kV. Samples were prepared by drop-casting micelle solutions onto carbon-coated copper grids and then air-drying at room temperature. The uranyl acetate (UA) was used as the negative staining. Furthermore, α-TOS and curcumin encapsulation and the loading ability of micelles were measured using UV-VIS spectroscopy at wavelengths of 286 nm and 430 nm, respectively. Encapsulation efficiency (EE) and drug-loading capacity (DL) were calculated using the following Equations (1) and (2), respectively:

(1) $$\mathrm{EE}\;\left(\%\right)=\frac{\mathrm{Weight}\;\mathrm{of}\;\mathrm{TOS}\;\mathrm{or}\;\mathrm{curcumin}\;\mathrm{in}\;\mathrm{the}\;\mathrm{micelle}}{\mathrm{Weight}\;\mathrm{of}\;\mathrm{TOS}\;\mathrm{or}\;\mathrm{curcumin}\;\mathrm{intial}\;\mathrm{feeding}\;}\times 100,$$

(2) $$\mathrm{DL}\;\left(\%\right)=\frac{\mathrm{Weight}\;\mathrm{of}\;\mathrm{TOS}\;\mathrm{or}\;\mathrm{curcumin}\;\mathrm{in}\;\mathrm{th}\;\mathrm{micelle}}{\mathrm{Weight}\;\mathrm{of}\;\mathrm{drug}\;\mathrm{loaded}\;\mathrm{micelle}}\times 100.$$

### 2.4. In Vitro Drug Release

An in vitro drug release study was done using dialysis methods. Briefly, 2 mL of TOS/curcumin-loaded micelle solution (at curcumin and TOS concentrations of 0.955 mg/mL and 1.7 mg/mL, respectively) were transferred to a dialysis bag (12–14 kDa) and then dialyzed in 10 mL of phosphate buffer solution at pH 7.4 in the presence and absence of 5 mM GSH under stirring at 37 °C. Aliquots (1 mL) were taken from the released medium at a predetermined time and the same aliquot of blank PBS with and without GSH was added back to keep the volume constant. The absorbance of TOS and curcumin was measured at 286 nm and 430 nm wavelengths, respectively. The TOS and curcumin concentrations were calculated based on the standard curve, and the percentage of accumulated drug release was plotted against time.

### 2.5. In Vitro Cytotoxicity

The in vitro cytotoxicity of free TOS, free curcumin, and drug-loaded micelles were evaluated against PAN02 pancreatic cancer cells using MTT assay. Nanoformulated TOS or curcumin was dissolved in PBS buffer (pH 7.4), whereas the free TOS and curcumin was first dissolved in the DMSO and then diluted using PBS buffers. The final sample solution was prepared using medium before treating cells according to the mentioned concentration. Briefly, PAN02 pancreatic cancer cells were seeded at a density of 5 × 10^3^ cells per well in 96-well plates and incubated for 24 h to allow cell attachment. Then, the cells were incubated in a concentration gradient at 37 °C with free TOS, free curcumin, and drug-formulated micelles (at concentrations of 100, 50, 25, 12.5, and 6.25 µg/mL for TOS and at curcumin concentrations of 50, 25, 12.5, 6.25, and 3.125 µg/mL). After 48 h of incubation, the medium was removed, and new medium with 20 μL MTT (5 mg/mL) was added and further incubated for 4 h. The medium in each well was removed, and 100 μL DMSO was added to dissolve the internalized purple formazan crystals. The absorbance was measured at the test wavelength of 492 nm using an enzyme-linked immunosorbent assay (ELISA) reader (Power Wave XS, BioTek, Winooski, VT, USA). The relative cell viability (%) was calculated using the following equation:

(3) $$\text{Cell}\;\text{viability}\;(\%)\;=\;\frac{\text{absorbance}\;\text{of}\;\text{test}\;\text{cells}\;\text{−}\;\text{absorbance}\;\text{of}\;\text{reference}}{\text{absorbance}\;\text{of}\;\text{controlled}\;\text{cells}\;\text{−}\;\text{absorbance}\;\text{of}\;\text{reference}}\;\times \;100$$

Similarly, the in vitro cytotoxicity effects of materials—PAH, PAH-Cit, PLGA-Cys, and PAH-SS-PLGA—were investigated using MTT assays as explained above after treating PAN02 pancreatic cancer cells at different doses. To investigate the activity of the TOS–curcumin combinations, a fixed ratio (TOS: Curcumin; 2:1 *w*/*w* ratio) of the two drugs was generated based on a ratio defined by the IC_50_ of TOS and curcumin. Then, these ratios were tested over a broad range of effective doses for activity against the PAN02 pancreatic cancer cells using the MTT assay as mentioned above. The dose-dependent effects of the drugs when used alone and in combination were analyzed using CompuSyn, a computer program that analyzes dose–response data according to the Chou and Talalay median effect principle [34]. The program generates combination index (CI) values from the dose–response curves and showed whether the interaction between the two drugs results showed synergistic (CI < 1), additive (CI = 1), or antagonistic (CI > 1) effects. CI was calculated as:

CI = [(D)1/(Dx)1] + [(D)2/(Dx)2];(4)

where (Dx)1, (Dx)2 = the concentrations of drug 1 and drug 2 that induced a 50% inhibition of cell proliferation. (D)1, (D)2 = the concentrations of drug 1 and drug 2 in combination that also inhibited cell growth by 50%. The CI index was calculated using CompuSyn.exe software (ComboSyn. Inc., Paramus, NJ, USA, 2005).

### 2.6. Colony Assay

The colony assay was evaluated to investigate the long-term effects of the free drugs and nanoformulated drugs using PAN02 pancreatic cancer cells. Briefly, the cancer cells were seeded at a density of 500 well in 6-well plates and incubated for 24 h to allow cell attachment. Then, the cells were incubated in a concentration gradient at 37 °C with free TOS, free curcumin, and drug-loaded micelles (at concentrations of 1.25, 2.5, 5, 10, and 20 μg/mL and 0.312, 0.625, 1.25, 2.5, and 5 μg/mL for TOS and curcumin, respectively) for 6 days. Similarly, for the combination drug treatments, free TOS + free curcumin and dual drug-loaded micelles (at concentrations of TOS (curcumin): 0.625(0.3125), 1.25(0.625), 2.5(1.25), 5(2.5) and 5(10) μg/mL, the number in the brackets shows the concentration of curcumin, whereas that outside the bracket is for TOS). After 6 days of incubation, the medium was removed, washed with PBS, and 3 mL of methanol was added to fix the cell for 2 h. Then, methanol was removed, washed three times with PBS, and further incubated with 3 mL of 0.5% crystal violet solution for 3 h. The plate was washed with tap water until the background was clear and then air-dried. Finally, the plate was scanned using a Microtek scanner.

### 2.7. Apoptosis Assay

The apoptosis assay was detected using an Annexin V-Alexa Fluor 488 apoptosis detection kit using flow cytometry. Briefly, PAN02 pancreatic cancer cells were seeded into 6-well plates at a density of 1 × 10^5^ cells/well for 24 h and washed three times with PBS to remove dead cells. Free TOS, free curcumin, and drug-loaded micelles (at TOS and curcumin concentrations of 30 and 15 μg/mL, respectively) were incubated with cells for 48 h. Then, the cells were washed, collected, and Alexa Fluor@ 488 annexin V and PI were added according to the manufacturer’s recommendation with the samples prior to analysis. Cells that were propidium iodide (PI)-negative and Annexin V-negative are considered live cells, PI-negative and Annexin V-positive cells are considered early apoptotic, and cells that are positive to both PI and Annexin V are considered late apoptosis/necrotic cells.

### 2.8. Cell Cycle Analysis

Cell cycle analysis was detected using flow cytometry after PAN02 pancreatic cancer cells were treated with TOS, curcumin, and drug-loaded micelles. Briefly, cells (1 × 10^5^ cells/well) were seeded on 6-well plates and co-cultured with free TOS, curcumin, and drug-loaded micelles (at TOS and curcumin concentration of 30 and 15 μg/mL, respectively) for 48 h. Then, cells were trypsinized, washed with PBS, and fixed on ice-cold ethanol overnight. Finally, the cell suspension was centrifuged at 1000 rpm for 5 min, and the cell pellets were finely washed by PBS and stained by PI/Triton X-100/ RNA_ase_ solution for 30 min at 37 °C. A BD FACS Calibur flow cytometer and ModFit LT software were applied for cell cycle analysis.

### 2.9. Cellular Uptake Study

Curcumin and/or rhodamine-loaded micelle cellular uptake was investigated using PAN02 pancreatic cancer cells by a simple modification of our previous protocol [35]. Briefly, the cells were seeded, grown in a four-well plate at a density of 5 × 10^4^ cells/well, and incubated at 37 °C under 5% CO_2_ for 24 h. The cellular uptake of curcumin and/or rhodamine (at a curcumin and/or rhodamine concentration of 10 μg/mL)-loaded micelles was investigated by using Nikon Fluorescence microscopy after 3 h of incubation. The image was analyzed using NIS-Elements imaging software.

### 2.10. Statistical Analysis

All results are representative of at least three sets of independent experiments with samples performed in duplicate or triplicate in each experiment. The data were represented as the mean ± standard deviation. The significances of the differences were determined using Student’s *t*-test, one-tailed, for each paired experiment. * *p*-value < 0.05 was considered statistically significant in all cases. * *p* < 0.05, ** *p* < 0.01, *** *p* < 0.001.

## 3. Results and Discussion

### 3.1. Synthesis and Characterization of PLGA-Cys, PAH-Cit, and PAH-SS-PLGA Conjugates

**Figure 1:**
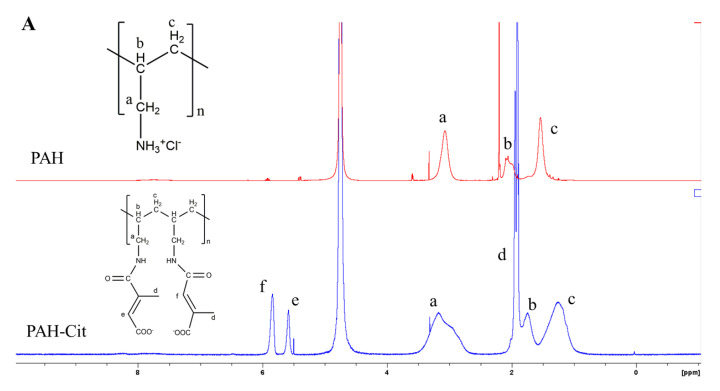

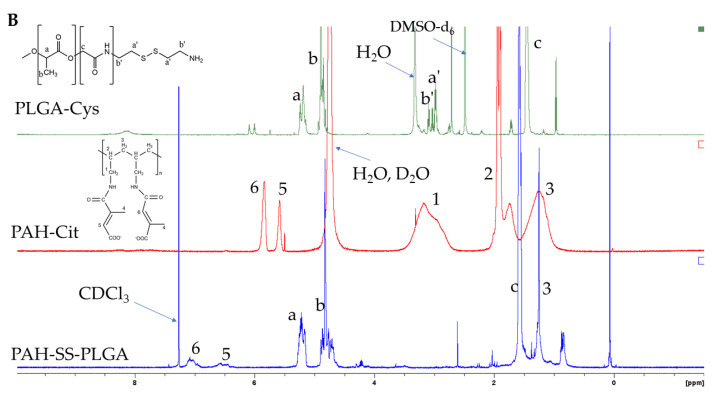
^1^H-NMR of (**A**) PAH and PAH-Cit and (**B**) PLGA-Cys, PAH-Cit, and PAH-SS-PLGA. Note: D_2_O was used as a solvent for PAH and PAH-Cit, whereas DMSO and CDCl3 solvent were used for PLGA-Cys and PAH-SS-PLGA, respectively. PAH: poly (allylamine hydrochloride), PAH-Cit: poly(allylamine)-citraconic anhydride, PLGA-Cys: poly(lactic-co-glycolic acid)-cystamine.

The PAH-SS-PLGA co-polymer was synthesized by conjugating the carboxyl group of PAH-Cit with an amino group of PLGA-Cys in the presence of EDC and NHS, as shown in Scheme 1. In this study, PAH-Cit was used as the backbone of the carriers, whereas cystamine were used as the GSH-sensitive segments. Furthermore, the hydrophobic segment of PLGA facilitates micelle formation to encapsulate the hydrophobic drugs, TOS and curcumin. The structures of PLGA-Cys, PAH-Cit, and PAH-SS-PLGA were confirmed using ^1^H-NMR and FTIR. In addition, UV-VIS was used to confirm TOS as well as curcumin loading and release from the PAH-SS-PLGA micelle. As shown in Figure 1A, the ^1^H-NMR peaks of free PAH that were observed at 1.2 ppm correspond to the methylene protons (-CH2-) on the PAH polymer backbone, those at 1.7 ppm correspond to the backbone methine protons, and the 3.20 ppm peak corresponds to the methylene protons adjacent to the amine group. After the conjugation of PAH with citraconic anhydride, additional peaks were observed at the chemical shifts of 1.9, 5.6, and 5.8 ppm, which confirmed PAH-Cit was successfully synthesized. Two isomers of PAH-Cit were obtained, with the methyl group either distal or proximal to the newly generated amide bond. The ratio of proximal to distal isomers was about 1.37. The degree of grafting (dg) of citraconic acid to PAH was approximately 46% by considering the integral area ratio of distal/proximal CH3 of Cit to CH2 group of PAH. Similarly, as shown in Figure 1B, PLGA was successfully conjugated with cystamine. The chemical shifts at 2.98 and 3.01 ppm were observed, which correspond to the different methylene (2H, –CH2–S–; 2H, –CH2–N) groups of the cystamine. Moreover, PAH-SS-PLGA was successfully synthesized by conjugating PAH-Cit and PLGA-Cys.

**Figure 2:**
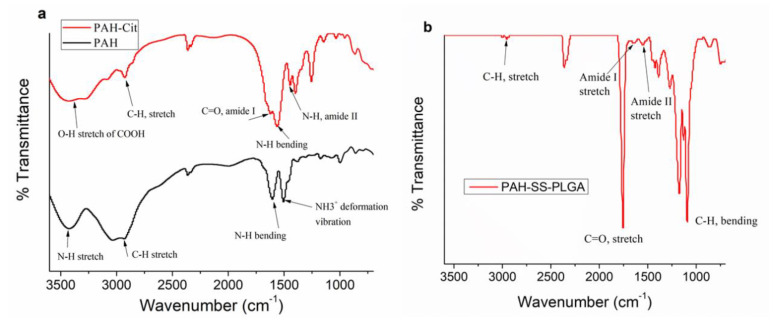
FTIR peak of (**a**) PAH, PAH-Cit, and (**b**) PAH-SS-PLGA.

In addition to ^1^H-NMR, the chemical structures of the PAH, PAH-Cit, and PAH-SS-PLGA were further confirmed using FTIR, as shown in Figure 2. All major characteristic peaks of PAH, PAH-Cit, and PAH-SS-PLGA were observed within the range of 4000−620 cm^−1^. Several major peaks were observed at approximately 3200–3364 cm^−1^ (N-H stretch), 2883−2915 cm^−1^ (C−H stretch), 1756 cm^−1^ (C=O, stretch), and 1320–1410 cm^−1^ (C−H bending). In addition, there are new peaks at 1619 cm^−1^ (amide I bond) and 1538 cm^−1^ (amide II bond), which confirm amide bond formation between PAH and citraconic anhydride and PAH-Cit and PLGA-Cys to form PAH-Cit and PAH-SS-PLGA, respectively. Overall, both ^1^H-NMR and FTIR results confirmed the successful synthesis of PAH-Cit and PAH-SS-PLGA.

### 3.2. Preparation and Characterization of Empty Micelles and Drug-Loaded Micelles

**Figure 3:**
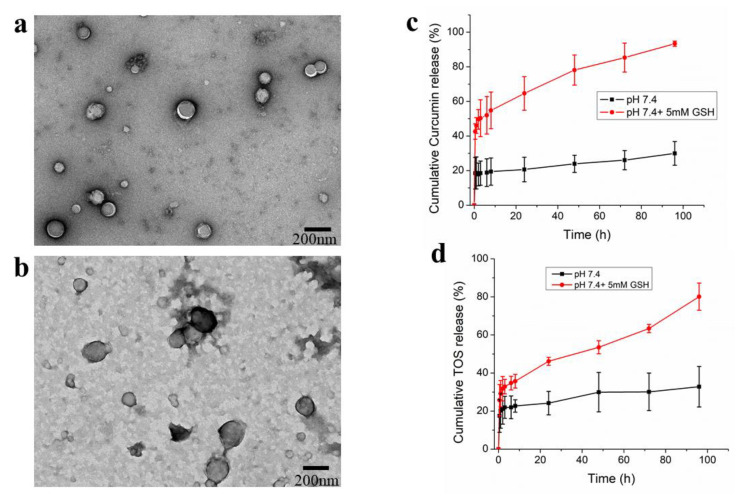
TEM image of PAH-SS-PLGA-micelle in the absence (**a**) and presence of 5 mM GSH (**b**), in vitro curcumin (**c**) and TOS (**d**) release from PAH-SS-PLGA micelle in the presence and absence of GSH (5 mM). Data are expressed as mean ± SD values (*n* = 3).

The PAH-SS-PLGA conjugate could self-assemble into micelles in aqueous solution due to its amphiphilic nature and was further used to encapsulate hydrophobic TOS and curcumin drugs via emulsion solvent evaporation methods. The mean particle size of TOS or curcumin-loaded micelles was 172.93 ± 1.1 nm and 194.17 ± 1.7 nm, respectively, with a low polydispersity index, indicating the formation of nanoparticles with a narrow and unimodal distribution, as shown in Table 1, based on the DLS measurement. The zeta potential results showed that both drug-loaded and empty micelles displayed positive surface charges of approximately 22 mV, which shows that the synthesized carrier is very stable due to charge stabilization (i.e., the electrostatic repulsive forces are high enough to prevent aggregation) [36]. The surface morphology of the synthesized micelle was observed to have a spherical shape, using TEM, as displayed in Figure 3a. Moreover, after incubating for 6 h with 5 mM GSH, the synthesized micelle slightly lost its spherical shape, which is due to the cleavage of disulfide bonds, which in turn leads to disassembly of the micelle, as shown in Figure 3b.

### 3.3. Drug Loading and Drug Release Study from PAH-SS-PLGA Micelle

The encapsulation efficiency (EE%) of the PAH-SS-PLGA micelle was assessed both for curcumin and TOS. The calculated EE% of the micelles was 85% and 95.5% for TOS and curcumin, respectively. Furthermore, to validate that GSH induced TOS/curcumin release from the micelle, the drug-release behavior was determined by a dialysis method in PBS buffer (pH 7.4) in the presence and absence of GSH (5 mM). The amount of TOS and curcumin released was measured by UV−VIS spectrophotometer at λmax of 286 nm and 420 nm, respectively. In vitro drug release study revealed that excess TOS and or curcumin release was observed in the presence of GSH (5 mM) at the physiological pH value, as shown in Figure 3c,d, showing that the synthesized PAH-SS-PLGA micelle is GSH sensitive.

### 3.4. In Vitro Cytotoxicity Studies

**Figure 4:**
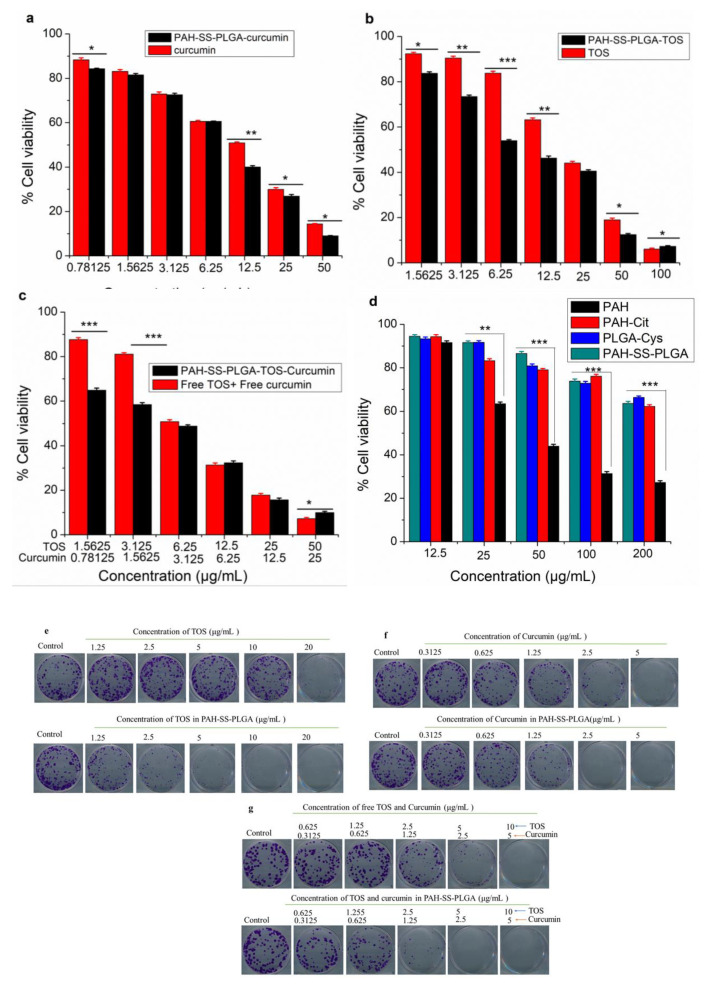
MTT in vitro cytotoxcity results using PAN02 pancreatic cancer cells (**a**) free curcumin and curcumin-loaded micelles, (**b**) free TOS and TOS-loaded micelles, (**c**) a combination of TOS and curcumin, (**d**) PAH, PAH-Cit, PLGA-Cys and PAH-SS-PLGA, and (**e**–**g**) representative images of the crystal violet-stained colonies using PAN02 pancreatic cancer cells at different doses of free TOS and PAH-SS-PLGA-TOS, free curcumin and PAH-SS-PLGA-curcumin, and a combination of free TOS and curcumin and nanoformulated TOS/curcumin, respectively. Data are expressed as mean ± SD values (n = 3). (* *p* < 0.05, ** *p* < 0.01, *** *p* < 0.001).

Dependent on the drug release mechanism or their direct interaction with the cell of interest, nanoformulated drugs show different anti-cancer activity in the in vitro cytotoxicity study in comparison to free drugs. Hence, in order to investigate the in vitro cytotoxicity effect of free TOS, free curcumin, TOS + curcumin, or TOS/curcumin-loaded micelles, cell viability was evaluated by the MTT assay using PAN02 pancreatic cancer cells. As shown in Figure 4a,b, free TOS, free curcumin, and TOS/curcumin-loaded micelles could inhibit the growth of PAN02 cancer cells in a concentration-dependent manner. Most interestingly, nanoformulated drugs showed higher cytotoxicity than the free drugs in Figure 4c, which is maybe due to the greater accumulation of nanoformulated drugs via endocytosis and slow drug release mechanism, which will prevent the drugs’ efflux. Similarly, the cytotoxicity of the materials (PAH, PAH-Cit, and PAH-SS-PLGA) was also investigated, as shown in Figure 4d. The result revealed that PAH shows higher cytotoxicity at the tested higher concentration in comparison to its derivative (PAH-Cit and PAH-SS-PLGA). This is due to the cationic nature of PAH, which will disrupts the integrity of the cellular membranes. It was reported that substitution of the amino groups of PAH (such as guanidinylation, glycolylation, or imidazolyl) significantly reduces its cytotoxicity [22,23]. Hence, it is expected that surface modification of the PAH polymer with citraconic anhydride and PLGA-Cys could also minimize the PAH-related cytotoxicity.

The colony assay is the other alternative assay to monitor a cancer cell’s ability to produce a viable colony after treatment. The colony-forming assay gives information about a long-term proliferative potential of cells that cannot be determined by short-term assays. Hence, combining the colony-forming assay with assays that measure cell death or cell viability in short time periods is likely to offer more information about the fate of cells in a population. Hence, in this study, colony formation assay was done to investigate the long-term anti-cancer potential of free TOS, curcumin, or nanoformulated TOS and curcumin using PAN02 pancreatic cancer cell lines. As shown in Figure 4e–g, the colony formation results revealed an improved therapeutic efficacy of nanoformulated TOS and or curcumin than free TOS and or curcumin at a lower dosage. The enhanced anti-cancer therapeutic efficacy of nanoformulated TOS and/or curcumin compared to free TOS or curcumin is due to the slow release of TOS or curcumin from internalized nanocarriers in PAN02 pancreatic cancer cells. Together with MTT assay, this result indicates that nanoformulated drugs have better anti-proliferative actions against PAN02 pancreatic cancer cells.

The combination effects of the TOS and curcumin were also investigated for PAN02 pancreatic cancer cells, as shown in Figure 4c. As shown above, a combination of TOS and curcumin as well as dual-loaded micelle shows better therapeutic efficacy than the free drugs alone. In addition, the dose-dependent effects of the drugs when used alone and in combination were analyzed using CompuSyn, which is a computer program that analyzes dose–response data according to the Chou and Talalay median effect principle [34]. The program generates combination index (CI) values from the dose–response curves and provides an indication as to whether the interaction between the two drugs results in synergistic (CI < 1), additive (CI = 1), or antagonistic (CI > 1) effects. As shown in Table 2, at all tested concentrations, the calculated CIs value were less than one, indicating synergistic effects for all nanoformulated-drugs (PAH-SS-PLGA-TOS-Curcumin), whereas at lower concentrations, the combination of free TOS + free curcumin elicited an antagonistic effect on the cellular proliferation inhibition of PAN02 pancreatic cancer cells.

### 3.5. Apoptosis and Cell Cycle Analysis

**Figure 5:**
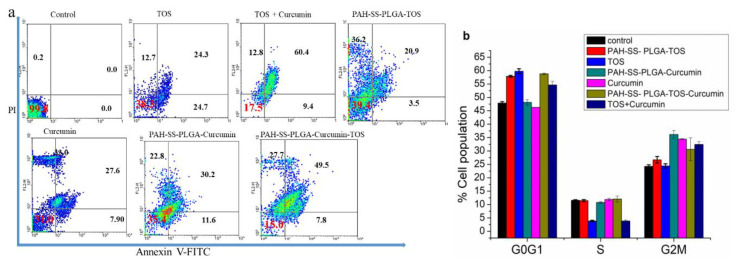
(**a**) Apoptosis assay of free TOS, free curcumin, free TOS + free curcumin, and TOS/curcumin-loaded micelles and (**b**) cell cycle analysis of free TOS, free curcumin, and TOS/curcumin-loaded micelles using PAN02 pancreatic cancer cells. Note: The concentration of free TOS and TOS in the micelles was 30 μg/mL, whereas the concentration of free curcumin and curcumin in the micelles was 15 μg/mL. In addition, for the combination treatments, 30 μg/mL and 15 μg/mL were used together for TOS and curcumin, respectively. Data are expressed as mean ± SD values (n = 3). (* *p* < 0.05, ** *p* < 0.01, *** *p* < 0.001).

Apoptosis is the other key factor that accompanies cancer cell death in chemotherapy. The induction of apoptosis can be recognized by several molecular mechanisms (including the activation of caspases and cleaved poly (ADP-ribose) polymerases (cPARP) or a change of cellular morphology [37]. The apoptotic effect of free TOS, curcumin, and drug-loaded micelles against PAN02 cancer cells was investigated using an annexin V and PI assay. As shown in Figure 5a, an increase in apoptosis of PAN02 cancer cells was observed after treatment with free TOS, curcumin, and nanoformulated drugs for 48 h. The percentages of necrosis, early, and late apoptotic cells among those treated with free TOS (30 μg/mL) were 12.7%, 24.3%, and 24.7% respectively, whereas the figures were 0.2%, 0%, and 0% in untreated cells, respectively. The percentages of necrosis, early, and late apoptotic cells among those treated with nanoformulated TOS (at a TOS concentration of 30 μg/mL) were 36.2%, 20.9%, and 3.5%, respectively. Similarly, the percentages of necrosis, early, and late apoptotic cells among those treated with free curcumin (15 μg/mL) were 33.0%, 27.6%, and 7.9% respectively, whereas the figures were 22.8%, 30.2%, and 11.6% for nanoformulated curcumin, respectively. Most interestingly, the percentages of early and late apoptotic cells among those treated with combination drugs or free curcumin and free TOS were 60.4% and 9.4%, respectively, while those of nanoformulated curcumin–TOS were 49.5% and 7.8% (at curcumin and TOS concentration of 15 μg/mL and 30 μg/mL, respectively). Similarly, several researchers reported that curcumin can induce apoptosis and subsequently contribute to cancer cell death [38,39]. In addition, proapoptotic activities of TOS are also reported in cancer cells via mitochondrial inhibition, the most effective mitocans in inducing the apoptosis, and the production of superoxide radicals [40,41,42]. Collectively, these results suggested that a co-treatment of curcumin and TOS would enhance the apoptotic cell death of PAN02 pancreatic cancer cells.

Furthermore, investigation of cell cycle distribution and cell proliferation is essential for studying cell growth differentiation, apoptosis, and senescence. During cell cycle progression, there are variations in the genetic content, and proliferating cells consecutively undergo different stages, such as G0G1 (preparation for DNA synthesis), S (DNA synthesis), and G2M phases (preparation of cell division and mitosis), which can be observed by fluorescent dye, such as propidium iodide (a fluorescent DNA-intercalating dye), using flow cytometry. In this study, the cell cycle analysis was carried out for PAN02 pancreatic cancer cells after treating with free TOS, curcumin, and TOS/curcumin-loaded micelles for 48 h. As shown in Figure 5b, PAN02 cells treated with free TOS and TOS + free curcumin showed a higher accumulation of cells in the G0G1, while TOS and or curcumin-loaded micelles showed cell inhibition at the G2M phase compared to that of the untreated PAN02 pancreatic cancer cells. A similar observation was observed that showed curcumin-mediated cell cycle arrest at G0G1 and G2M checkpoints [43].

### 3.6. Cellular Uptake Studies

**Figure 6:**
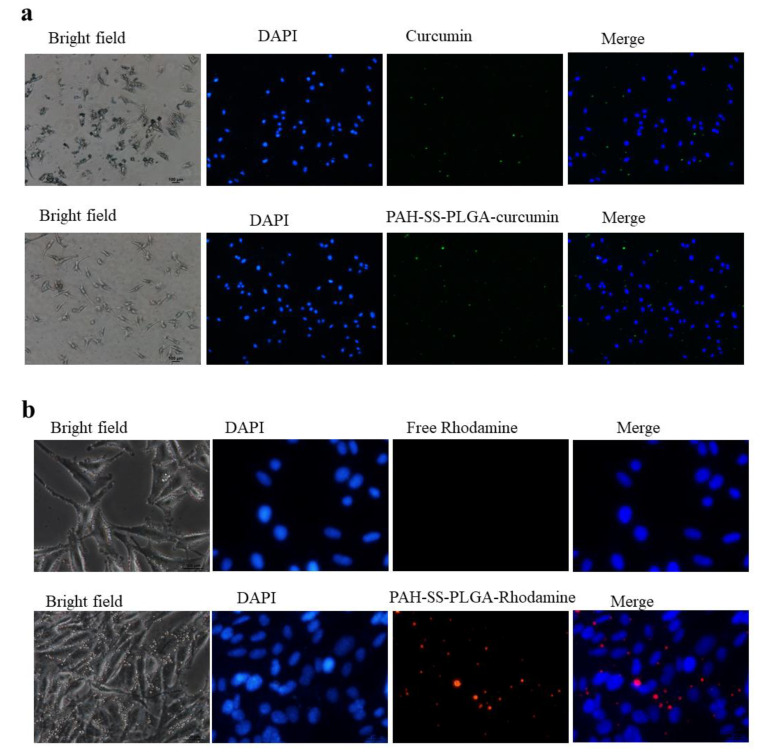
Cellular uptake of (**a**) free curcumin and curcumin-loaded micelles as well as (**b**) free rhodamine and rhodamine-loaded micelles using confocal microscopy after incubating for 3 h. Scalebar is 100 μm.

In drug delivery, the cellular uptake of nanoparticles by the cells of interest is a crucial step to obtain high therapeutic efficacy. Hence, understanding the cellular uptake and intracellular trafficking of nanoparticles is vital in designing biocompatible and efficient nanocarriers. This experiment was designed to observe the internalization efficacy of curcumin and curcumin-loaded micelles as well as that of rhodamine and rhodamine-loaded micelles by utilizing their inherent fluorescence property using confocal microscopy. As shown in Figure 6a,b, after 3 h of incubation, green and red fluorescence was observed in the cytoplasm, which confirms that curcumin and/or rhodamine-loaded micelles were up taken by PAN02 pancreatic cancer cells, respectively. In comparison to free curcumin or rhodamine, the improved uptakes were observed for the nanoformulated curcumin or rhodamine. This may be due to the endocytosis of nanoformulated curcumin and nanoformulated rhodamine compared to the passive diffusion of the drug itself. Moreover, the electrostatic interaction between the positive zeta potential of the PAH-SS-PLGA and the negative charge of the cellular membrane might also contribute to its enhanced cellular uptake. These characteristics could exert positive biological effects of nanoformulated curcumin and or TOS. Overall, the use of the PAH-SS-PLGA nanocarrier may be an attractive option for increasing the therapeutic efficacy of anti-cancer drugs.

## 4. Conclusions

A GSH-sensitive copolymer, PAH-SS-PLGA, was synthesized after the modification of highly cytotoxic PAH with citraconic anhydride and PLGA-Cys. The synthesized co-polymer has an amphiphilic nature and tends to encapsulate hydrophobic drugs, TOS and curcumin, in its core region during micelle synthesis in the aqueous medium. The drug release study revealed higher drug release in the presence of 5 mM GSH than at physiological pH value. Fluorescence microscopy images revealed that curcumin and or rhodamine-encapsulated micelles were efficiently taken up by PAN02 pancreatic cancer cells. The in vitro cytotoxicity results revealed that combination treatments enhanced anti-cancer therapeutic efficacy compared to free TOS or curcumin. In addition, at all tested doses, nanoformulated drugs showed synergistic effects, whereas antagonistic effects were seen at a lower dose combination of free drugs. Overall, a synthesized co-polymer would be a promising carrier for TOS/curcumin to enhance its therapeutic efficiency.

## Figures and Tables

**Table 1:** The mean particle size, polydispersity index (PI), and zeta potential of empty, TOS, and/or curcumin-loaded micelles in distilled water (dH_2_O) at 25 °C.

Sample Name	Particle Size (nm)	Polydispersity Index (PDI)	Zeta Potential (mV)
PAH-ss-PLGA micelle	161.73 ± 2.5	0.22 ± 0.02	22.73 ± 1.17
PAH-ss-PLGA-TOS	172.93 ± 1.1	0.13 ± 0.02	21.57 ± 0.84
PAH-ss-PLGA-Curcumin	174.17 ± 1.7	0.16 ± 0. 02	19.77 ± 1.42
PAH-ss-PLGA-Curcumin-TOS	201 ± 2.1	0.21 ± 0.01	17.57 ± 0.54

**Table 2:** Effects of combined TOS and curcumin treatment on PAN02 pancreatic cancer cells. CI: combination index.

Drugs	Concentration of Drugs (μg/mL)	% Cell Viability	CI Value	Effects
Free TOS + Free Curcumin	50 + 25	7.23 ± 0.053	0.40125	synergism
	25 + 12.5	17.81 ± 0.028	0.53959	synergism
	12.5 + 6.25	31.34 ± 0.049	0.56649	synergism
	6.25 + 3.125	50.85 ± 0.039	0.65387	synergism
	3.125 + 1.5625	81.12 ± 0.115	1.48333	antagonistic
	1.5625 + 0.78125	87.63 ± 0.087	1.28316	antagonistic
PAH-ss-PLGA-TOS + PAH-ss-PLGA-Curcumin	50 + 25	9.95 ± 0.016	0.68485	synergism
	25 + 12.5	15.68 ± 0.028	0.61559	synergism
	12.5 + 6.25	32.35 ± 0.020	0.89538	synergism
	6.25 + 3.125	50.17 ± 0.044	0.97939	synergism
	3.125 + 1.5625	58.42 ± 0.039	0.76110	synergism
	1.5625 + 0.78125	64.89 ± 0.069	0.52037	synergism
